# Necrotizing fasciitis of the vulva due to carbapenem-resistant *Enterobacteriaceae* as a complication of acute myeloid leukemia treatment: a case report

**DOI:** 10.1186/s13256-021-03179-5

**Published:** 2022-04-11

**Authors:** Marta Leal Bento, Leonor Vasconcelos de Matos, Lídia Alves Ribeiro, Olavo Gomes, Filipa Nogueira, Graça Esteves, Sara Valle, Helena Martins, João Raposo

**Affiliations:** 1 Department of Hematology and Bone Marrow Transplantation, Centro Hospitalar Universitário de Lisboa Norte, Lisbon, Portugal; 2 Department of Oncology, Centro Hospitalar Lisboa Ocidental, Lisbon, Portugal; 3 Department of General Surgery, Centro Hospitalar e Universitário de Lisboa Norte, Lisbon, Portugal

**Keywords:** Carbapenem-resistant *Enterobacteriaceae*, Necrotizing fasciitis, Acute myeloid leukemia, Carbapenem-resistant *Klebsiella*, Hematology–oncology, Case report

## Abstract

**Background:**

Carbapenem-resistant *Enterobacteriaceae* strains have been reported in healthcare facilities with a rising incidence and are a major concern owing to infections that are often severe and can be potentially fatal, with limited therapeutic options. *Klebsiella pneumonia* represents the most frequently isolated microorganism.

**Case presentation:**

We report the case of a Caucasian 52-year old Caucasian woman with acute myeloid leukemia was admitted to the inpatient hematology unit at a university referral hospital in Portugal. This hospital has endemic colonization of Carbapenem-resistant *Enterobacteriaceae* and contention measures are being implemented to reduce spreading of these multidrug resistant bacteria. After receiving first line chemotherapy according to the intermediate-dose cytarabine regimen, in context of deep medullary aplasia, the patient developed a localized infection of the vulva, which progressed to a necrotizing fasciitis. This is a rare, life-threatening, and fulminant infection. Carbapenem-resistant *Klebsiella* was isolated in both vulvar exudate and blood cultures. The patient underwent multiple schemes of antimicrobials, but progressed with multiorgan compromise and was admitted to the intensive care unit for a short period for stabilization. Surgical debridement was performed twice with clinical improvement and, after 6 weeks, a skin graft was executed with good response. Reevaluation of the hematologic disease showed a complete response to first cycle of induction therapy. Despite success in resolving this complex infection, decisions regarding antibiotic treatment represented a tremendous challenge for the whole team. The importance of multidisciplinary collaboration was key for the patient’s recovery and survival, and therefore, needs to be acknowledged.

**Conclusions:**

This clinical case raises awareness on a clinical entity that can be life threatening and, therefore, requires a high level of suspicion to assure an early integrated approach to avoid complications. Endemic spreading of carbapenem-resistant *Enterobacteriaceae* is becoming a reality, and health policies need to be urgently undertaken at the national level to decrease morbidity and mortality because of health facilities-related infections.

## Background

Carbapenem-resistant strains have emerged among species belonging to the *Enterobacteriaceae* family. Carbapenemases are a class of enzymes that can confer resistance to carbapenems and other β-lactam antibiotic drugs, although not all carbapenemase-producing isolates are carbapenem resistant [1]. Several outbreaks caused by carbapenem-resistant *Enterobacteriaceae* (CRE) have been recorded in healthcare facilities around the world [2, 3] and in some places, CRE have become endemic [4]. In Portuguese healthcare institutions, the incidence of colonization and infection by CRE is rising and represents a major concern at a national level [5]. Serious concurrent conditions, for example, malignancy [6] and prior use of fluoroquinolones, carbapenems, or broad-spectrum cephalosporins have been independently associated with acquisition of infections caused by these strains [7]. In our hematology department of a university hospital, we are documenting an average of 30% colonization with CRE, placing the department as an endemic health facility for this specimen; this is vastly different from data from many European countries, which show low frequencies of CRE. Indeed, the European survey on carbapenemase-producing *Enterobacteriaceae* (EuSCAPE) project showed low frequencies of CRE in many European countries, with an average of 1.3 patients per 10,000 hospital admissions [8]. Therefore, we are implementing contention measures, with study and isolation of colonized patients to reduce spreading of these multidrug-resistant bacteria [5].

The sustained acquisition of antibiotic resistance poses a relevant and concerning public health issue. The alarm is justified by an increasing difficulty in treating CRE infections due to resistance to virtually all beta-lactam antibiotics, and often presenting additional mechanisms of resistance against second-line antibiotics such as aminoglycosides and fluoroquinolones. Moreover, recent studies have also shown emerging resistance to broad spectrum antibiotics (i.e., tigecycline and colistin), leaving very few therapeutic options [9].

Several studies have provided data regarding clinical outcomes for CRE infections. A systematic review extracted and analyzed the data from published studies and clinical cases of CRE infections, concluding that most of these patients had clinically severe infections (78% needing intensive care unit) and microorganisms were largely represented by *Klebsiella pneumoniae* (89%), followed distantly by *Pseudomonas* spp., *Escherichia coli*, *Serratia marcescens*, and *Enterobacter cloacae*.

The most common site of isolation was blood (52%), followed by lung (30%), and urine (10%) [8]. In the face of this critical scenario, and to raise awareness among healthcare professionals and public health policymakers, reporting critical clinical cases facing serious challenges of everyday practice is, in our view, not only helpful and educational, but also a responsibility. Here, we present a clinical case of a severe infection with necrotizing fasciitis from a carbapenem-resistant *Klebsiella*. To the best of our knowledge, although reports of necrotizing fasciitis caused by multiresistant extended spectrum beta-lactamase (ESBL)-producing *Enterobacteriaceae* have been published [10], there is no published data of a clinical case of necrotizing fasciitis with isolated carbapenem-resistant *Klebsiella* as a causative microorganism.

## Case presentation

We describe the case of a Caucasian 52-year-old woman, teacher, married. She was diagnosed with acute myeloid leukemia (AML) in January 2019, after presenting to the emergency department due to respiratory symptoms and fever. She was initially diagnosed with an acute respiratory tract infection, new-onset pancytopenia, and febrile neutropenia. After admission to the hematology inpatient unit for treatment and further investigation, a bone marrow aspirate and core biopsy revealed a hypercellularity with 42% myeloblasts, suggesting AML with maturation. Immunophenotyping showed 36.73% myeloblasts, CD34+, CD117+, and CD13+. Screening for gene rearrangements and mutations showed no recurrent mutation (FLT3, CEBPA, RUNX1, TP53 negative) and conventional cytogenetic analysis showed a normal karyotype.

This patient had previous medical history of essential hypertension and cholecystectomy, but took no chronic medication and there were no known allergies. There was no history of exposure to chemicals, alcohol, or tobacco. She had one successful pregnancy resulting in a healthy child with whom she lives in a rural area of Portugal together with her husband.

On examination, the patient appeared fatigued. Her temperature was 38.3 °C, blood pressure 137/77 mmHg, pulse 84 beats per minute, respiratory rate 16 breaths per minute, and she had an oxygen saturation of 97% on room air. The conjunctiva and oral mucosa were pale. She was eupneic at rest, without signs of breathing difficulty. Jugular vein was normally distended. Pulmonary auscultation showed normal breathing sounds. The abdomen was soft and nontender, and there was no hepatosplenomegaly. No peripheral adenopathies were identified. Neurologic examination showed no abnormality, namely, the patient had a Glasgow Coma scale of 15 points; naming and repetition was intact and fluent, she followed the 3-step commands, there was no impairment in the function of the cranial nerves, motor, or sensory function, and without asymmetries. There was normal finger-to-nose and heel-to-shin movement, and no tremor nor dysmetria were observed. She had a bilateral symmetric flexor plantar response, with no Hoffman’s or clonus. Her gait was normal.

Laboratory hemogram showed the following: hemoglobin 9.4 g/dL; leukocytes 1940 cells/µL; neutrophils 230 cells/µL; lymphocytes 1650 cells/µL; eosinophils 0 cells/µL; basophils 0 cells/µL; monocytes 60 cells/µL; platelets 61,000 cells/µL. Blood levels of glucose, phosphorus, magnesium, calcium, alkaline phosphatase, alanine aminotransferase, and total and direct bilirubin were normal, as were results of renal function and thyroid function tests. Serologies with electrochemiluminescence immunoassay (ECLIA) assay for human immunodeficiency virus (HIV) types 1 and 2, *Treponema pallidum* antibody, hepatitis B virus, hepatitis C virus, hepatitis A virus, hepatitis E virus, toxoplasmosis, and herpes virus type 2 were negative; serologies for cytomegalovirus, Epstein–Barr virus, and herpes virus type 1 were positive; and cytomegalovirus and Epstein–Barr viral loads were negative. Protein electrophoresis showed marginal changes in alfa1, beta1, and beta2 fractions. Gamma fraction was normal. Colonization for carbapenem-resistant *Klebsiella pneumoniae* was investigated with a rectal swap and was positive.

She received her first line of chemotherapy (CH) according to the Idarubicin in combination with cytarabine (IDAC) regimen (idarubicin intravenous 12 mg/m^2^/day for 3 days and cytarabine intravenous 100 mg/m^2^/day for 7 days) on 31 March 2019. As per protocol, the patient was started on prophylaxis for fungal and viral infections with oral fluconazole 200 mg/day and oral acyclovir 200 mg every 12 hours (q12h), and aggressive intravenous hydration was administered. On day +8, in deep medullary aplasia, a distal thrombophlebitis of the right lower limb with cutaneous bacterial infection was diagnosed and large spectrum antimicrobials were delivered: intravenous vancomycin 1 g loading dose and 1 g q12h, intravenous meropenem 2 g every 8 hours (q8h), intravenous amikacin 1.5 g every 24 hours (q24h), and intravenous colistin 9 M units loading dose and 4.5 M units q12h, with resolution. Three sets of blood cultures were collected, each set corresponding to an aerobic and an anaerobic culture medium flask BacT/Alert®. The samples were obtained by venipuncture in different sites and with a sterilized procedure. The protocol was repeated ate 24 hours but no infectious agent was identified.

**Fig. 1.**
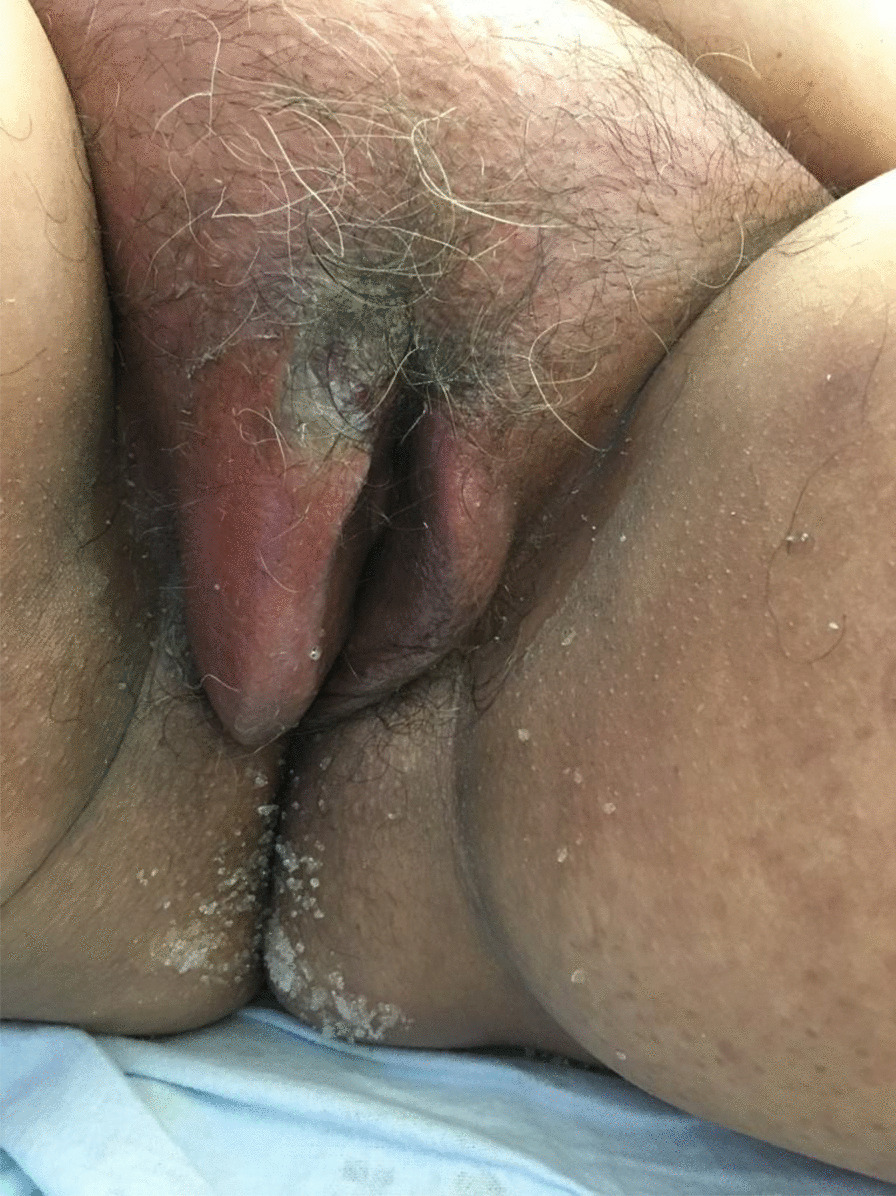
Vulvar phlegm in the superior border of major labia

**Fig. 2.**
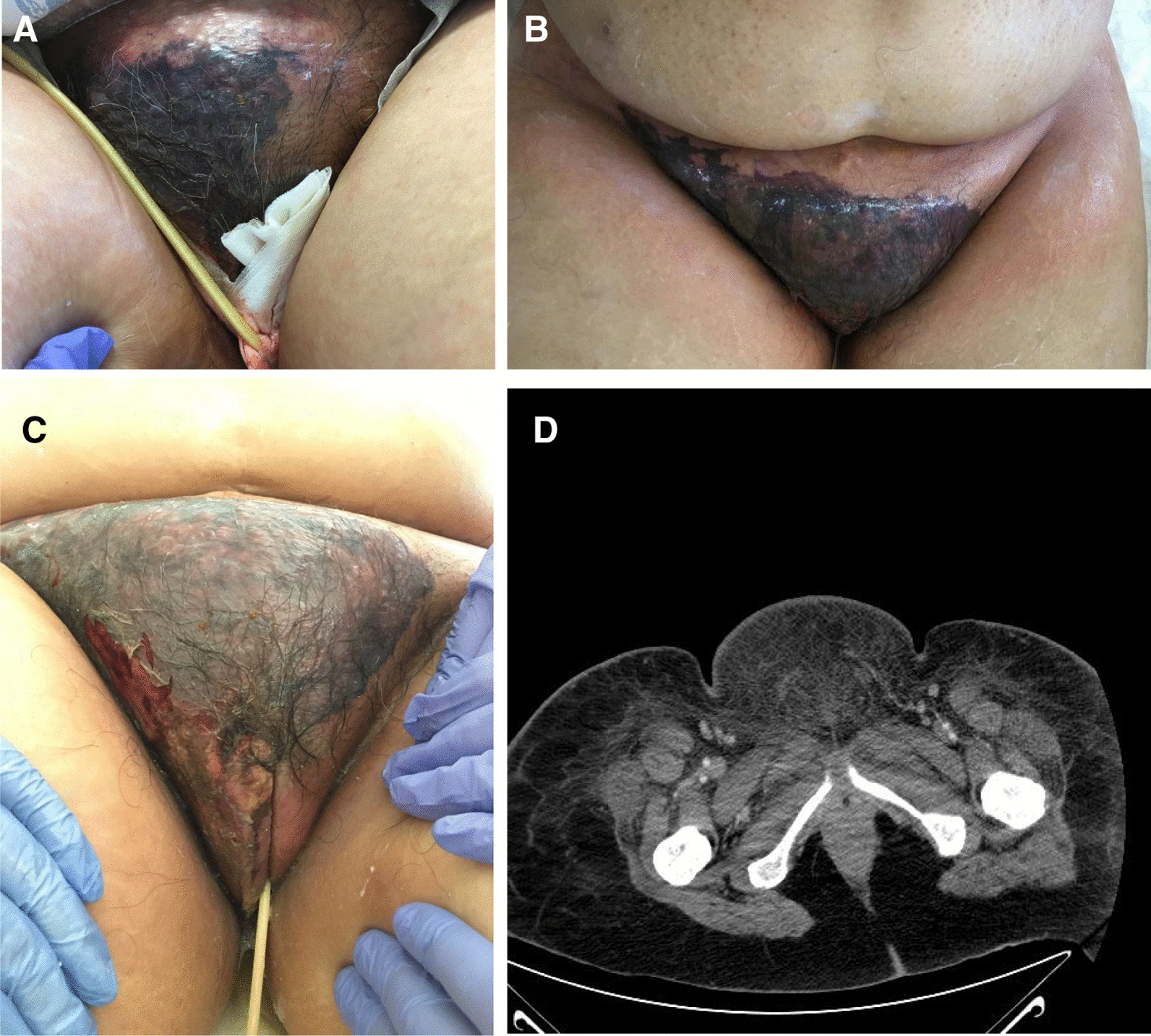
**A** Necrotizing fasciitis with extensive vulvar and pubic area necrosis, surrounding cellulitis and tissue edema. **B**, **C** Pubic area necrosis with loss of tissue. **D** Pelvic CT performed at day +12 after CH that shows diffuse perineal adipose tissue densification with cutaneous thickening and liquid lamination. These characteristics extend to the anterior inferior abdominal wall and presacral area. No areas of gas or liquid collected are visible. Mesenteric and iliac adenomegalies are present, the largest of 1.8 cm in the inguinofemoral right chain

On day +11 after CH, she developed a fever, accompanied by a rise in inflammatory parameters (C-reactive protein 30 mg/mL, procalcitonin 16 ng/mL). At observation, vulvar pain, swelling, and pruritus were present and a hard and painful phlegm could be seen in the right outer labia of the vulva, with exuberant surrounding inflammatory signs (Fig. 1). After multidisciplinary discussion, previous antimicrobials were suspended, except colistin and empirically started intravenous acyclovir 350 mg q12h, intravenous tigecycline 100 mg loading and 50 mg q12h, and intravenous daptomycin 500 mg q12h. The lesion rapidly progressed in the following 48 hours, with extensive cellulitis of the perineal area and necrosis of affected tissues, accompanied by hematologic, neurologic, liver, and respiratory dysfunction (Fig. 2A–C). Again, three sets of blood cultures were collected, each set with an aerobic and an anaerobic flask following the procedure described previously, and vulvar exudate was collected with a swab. On blood cultures and vulvar exudate analysis, carbapenem-resistant *Klebsiella pneumoniae* was isolated, with resistance to meropenem and amikacin with minimal inhibitory concentration (MIC) superior to 16 and sensitivity to tigecycline and colistin. At this point, antibiotherapy was changed to intravenous ceftazidime/avibactam 2 g/0.5 g q8h, intravenous meropenem 2 g q8h, intravenous amikacin 1.5 g q24h, intravenous vancomycin 1 g q12h, and prophylactic oral acyclovir 200 mg q12h and oral posaconazole 400 mg q8h. This antibiotic regime was maintained for 13 days.

**Fig. 3.**
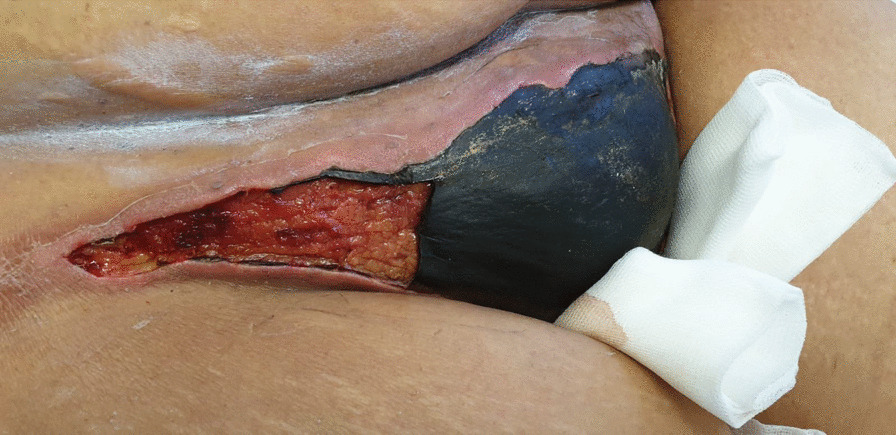
After return from the intensive care unit, viable tissue was present under the necrotized dried tissue with mummification

**Fig. 4.**
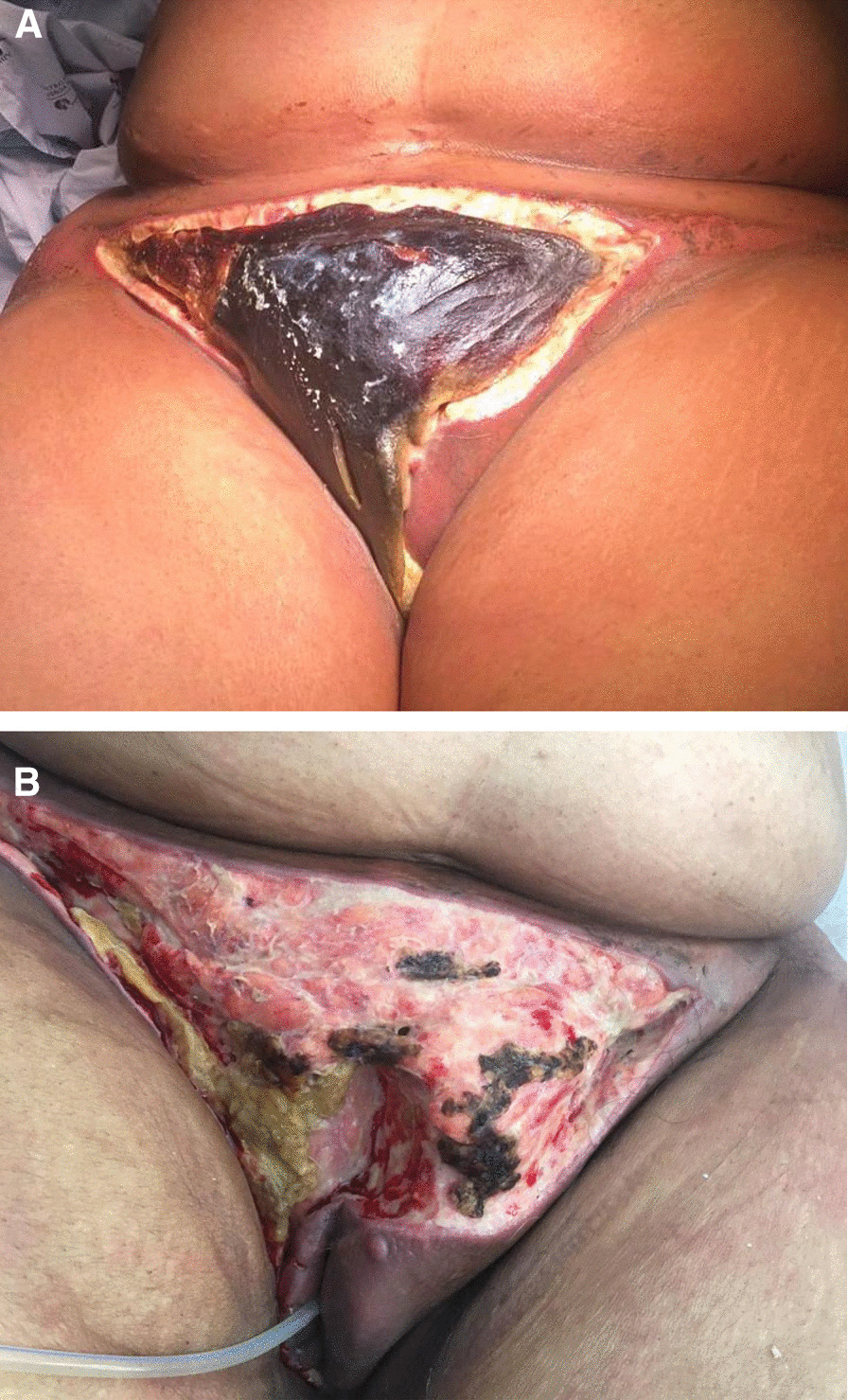
**A** Presurgical necrotic tissue with mummification of pubic area and underlying granulation and serous exudates. **B** Vulva and pubic area of the patient after surgical debridement. Large extension of the right labia majora was removed, viable tissue was present with no sign of infected exudates

A pelvic computed tomography (CT) scan was performed, showing no liquid or air collections (Fig. 2D). The surgical team performed superficial debridement of the soft tissue above the fascia of the pubic area extending down towards the right paragenital area. Postsurgery, the patient was admitted to the intensive care unit (ICU) due to maintained multiorgan dysfunction for a short period of time. At this point, the patient maintained vulvar necrotic tissue with less edema and cellulitis (Fig. 3) but granulation tissue, serous exudate, and mummified pubic wound developed (Fig. 4A). Local infection control was partial with systemic inflammatory response.

The surgical team performed new debridement with extensive removal of necrotic and granulation tissue (Fig. 4B). Simultaneously, antibiotics were switched to intravenous colistin 9 M units loading dose and 4.5 M units q12h, intravenous daptomycin 500 mg q12h, and intravenous amphotericin B 200 mg q24h, while maintaining meropenem and amikacin; ceftazidime/avibactam and vancomycin were suspended. In the removed skin it was possible to isolate a carbapenemase-producing *Klebsiella*. After the surgery and the last change of antibiotics, a rapid recovery was observed with progressive closure of the surgical wound and continued efforts in sterilization and cleansing of the exposed tissue, and with the support of pain team that implemented intensive anesthetic control.

Meanwhile, we evaluated the hematological response to the induction on day +44 after CH. Bone marrow aspirate showed 9% blastic cells and 37% monocytoid cells, considered blastic equivalents, but the immune phenotype evaluation revealed a blastic population of 3.3% of the total cellularity. Therefore, the patient was considered in complete response after the first cycle of induction therapy. Afterwards, we decided to start 5-azacitidine until more intensive chemotherapy was possible.

**Fig. 5.**
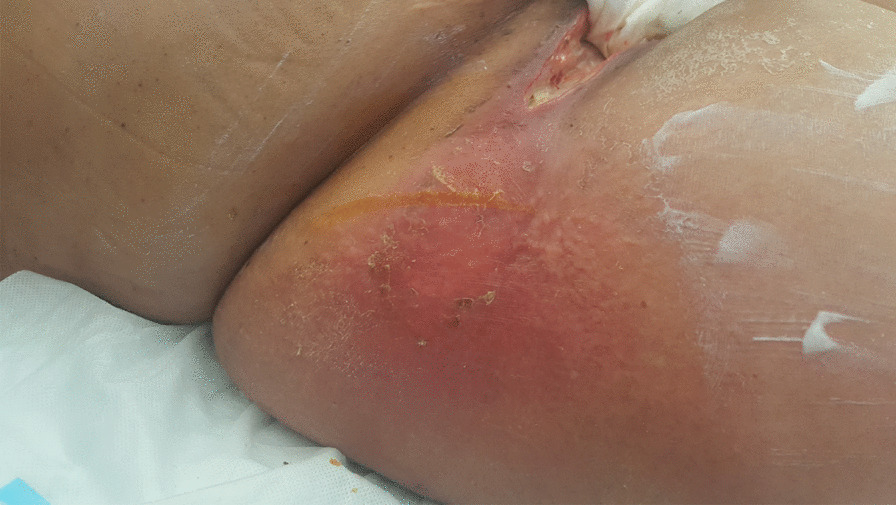
Cellulitis of the right border of surgical wound extended to the lateral faces of the thigh

On day seven after surgery, recurrence of local infiltration with cellulitis of the surgical borders and increased inflammatory C-reactive protein motivated new antibiotic rotation in an attempt to control bacteria from spreading (Fig. 5). While intravenous ceftazidime/avibactam 2 g/0.5 g q8h was resumed, colistin was suspended, as this remains an efficient weapon against multidrug-resistant Gram-negative bacteria such as carbapenemase-producing *Enterobacteriaceae* [11]. Progressive local and systemic control of infection was observed after ceftazidime/avibactam introduction.

Subsequently, the surgical wound continued to receive sterilization and cleansing with Dakin’s solution, hydrogen peroxide, and the application of honey antimicrobial wound matrix [12]. To ensure proper sterilization, negative pressure wound vacuum suction dressings were used with successful recovery of the wound with viable granulation tissue.

**Fig. 6.**
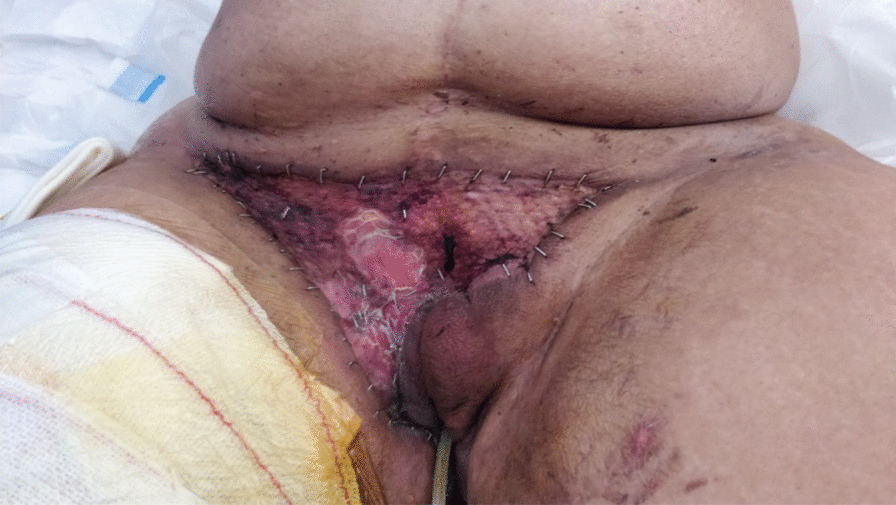
Vulva after surgical procedure with skin grafting

**Fig. 7.**
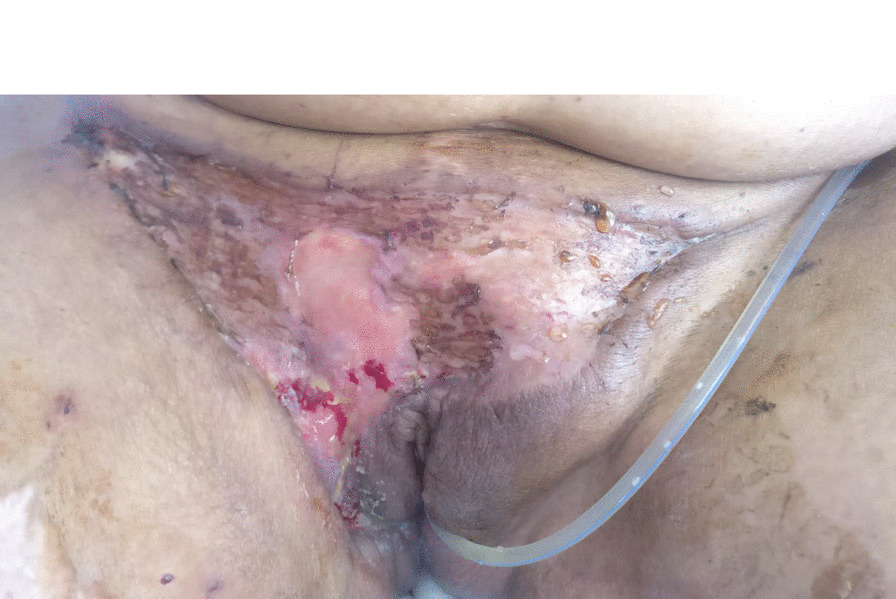
Vulva 2 weeks after surgical procedure with skin grafting

Finally, a skin graft was performed 6 weeks after surgical debridement of the vulva and pubic area, using donor skin from the patient’s right thigh. (Fig. 6) The procedure was executed by the plastic surgery team that maintained follow-up with regular application of hydration dressings that ensured good clinical evolution. (Fig. 7)

Meanwhile, antimicrobial therapy was suspended after a 27-day course of ceftazidime/avibactam and maintained follow-up for 2 weeks free from antibiotics, with no evidence of recurrence of infection.

On day 60 after performing the skin graft, the patient is asymptomatic, undergoing motor rehabilitation with excellent tolerance to exercise and excellent tissue healing.

Lastly, we underline the extreme relevance of the remission of the hematological disease in accomplishing infectious control and the necessary tissue healing.

In the context of a severe infectious complication with first line intensive chemotherapy, the patient was considered unfit for a new intensive regimen and maintained follow-up in the day hospital of hematology, undergoing cycles of 5-azacytidine 75 mg/m^2^/day for 7 days in 28-day cycles, with effective disease remission. At 6 months follow-up, the patient had 1.5% clonal blasts count in the bone marrow aspirate.

## Discussion and conclusions

Here we presented a case of a 52-year-old woman with a new onset diagnosis of acute myeloid leukemia with a severe infectious complication to first line intensive chemotherapy: a necrotizing fasciitis of the perineum from a multidrug-resistant bacteria, the carbapenemase-producing *Klebsiella*, which required a multidisciplinary and intensive approach to achieve successful treatment. To the best of our knowledge this is the first published case of a necrotizing fasciitis of carbapenemase-producing *Klebsiella pneumoniae* (CPKP) in a patient with acute myeloid leukemia.

Necrotizing fasciitis of the perineum is a rare, life-threatening, fulminant infection. Although the patient in this clinical case was a middle-aged woman, this condition is more common in elderly men. Many comorbidities have been associated with this infection, namely diabetes, chronic alcohol use, malignancy, morbid obesity, cardiovascular disorders, long-term corticosteroid treatment, and human immunodeficiency virus infection. All of these conditions have in common an immunosuppressive state that allows spreading of local bacteria into the perineal facial planes causing extensive tissue necrosis. It has been postulated that most of the bacteria originate from the urinary tract or from local intraabdominal or perineal infections such as abscesses, fistula, or vascular occlusions.

The approach to these patients includes immediate diagnosis, appropriate broad-spectrum antibiotics, and extensive surgical debridement to reduce mortality and morbidity, and to achieve local infection control. Delay in the diagnosis of Fournier gangrene leads to an extremely high death rate owing to rapid progression of the disease, leading to sepsis, multiple organ failure, and disseminated intravascular coagulation [13, 14].

In this clinical case, the patient was profoundly neutropenic, which provided increased susceptibility for the development of the infection, but neutropenia probably also protected the patient from the intensive neutrophilic infiltration of the tissues that is characteristic of Fournier gangrene. We might hypothesize that this protected the patient from further tissue necrosis and provided more time for surgical intervention on removal of the necrotic tissue.

The microorganisms causative of necrotizing fasciitis are considered virulent strains that promote the rapid spread of the disease in an immunocompromised host. *Enterobacteriaceae* belongs to this virulent species, together with Streptococcal, Staphylococcal, anaerobic organisms, and fungi. This one microorganism might produce the enzymes necessary to cause coagulation of the nutrient vessels, reducing local blood supply and, thus, tissue oxygen tension falls. The resultant tissue hypoxia allows for the growth of facultative anaerobes and microaerophilic organisms that can digest fascial barriers, fueling the rapid extension of the infection. Because several microorganisms are responsible for the pathology of disease, broad-spectrum antibiotics are necessary for local infection control, but without surgical removal of necrotic tissue it is not possible to treat the infection because the antibiotic does not reach the infected areas. Hematologic malignancies are a comorbid condition that can increase susceptibility for the development of necrotizing fasciitis due to the immunosuppressive state of the host. In 2013, D'Arena *et al*. produced a systematic review of all published cases of necrotizing fasciitis in patients with hematologic malignancies. They reported was that this infection was more common in acute myeloid leukemia patients, in particular acute promyelocytic leukemia, which was later seen as a possible complication of all-trans retinoic acid (ATRA) administration, a treatment able to induce high rates of complete remission. Patient characteristics were similar to those observed in other subgroups, with older age and male gender being more common. Regarding the involved agents, almost all cases reported were caused by *Pseudomonas aeruginosa*. The treatment approach and mortality rate did not differ from other subgroups [15].

The patient from the reported clinical case developed necrotizing fasciitis with an unusual microorganism for this population and no other infectious agents were isolated in the microbiological cultures, namely anaerobic microorganisms that are very frequently encountered in this setting. More so, because of the high antibiotic resistance profile of the bacterial agent isolated from cultures, as there are no reported cases of such an infection.

We believe this clinical case can help to shed some light into incredibly difficult clinical circumstances and to help guide the management of these patients. Since endemic spreading of CRE is becoming a reality in most countries, the authors are confident that more of these cases will emerge and want to contribute for the improved care for these patients.

With this case, we also emphasize the importance of a multidisciplinary care approach in these situations, which here involved a synergistic cooperation between the hematologic and surgical teams, together with the expertise of gynecology, infectious diseases, pharmacists, physiotherapists, nutritionists, and psychologists. Very interesting discussions were brought up during the patient’s treatment, which helped improve the approach and assure the best care. Of course, we also have to outline the tremendous challenge that this situation faced for the patient and her family: more than 2 months of contact isolation in an individual room, not being able to properly interact with loved ones, was very hard at all levels, and the efforts of all team members were crucial to assure adequate recovery of the patient, both physically and mentally.

We hope, with this case, to promote thinking and discussion of how to prevent these extremely distressing situations and also how to best manage and follow-up these patients. In this case, we highlight the need for a hematological remission, for continued surgical team care and follow-up, and antimicrobial coverage with study of microorganism’s sensitivity and concern for patient characteristics.

## Data Availability

The data that support the findings of this study are from the corresponding author, MLB, upon reasonable request.

## Abbreviations

AML: Acute myeloid leukemia
CRE: Carbapenem-resistant *Enterobacteriaceae*
CH: Chemotherapy
ATRA: All-trans retinoic acid
EuSCAPE: European survey on carbapenemase-producing *Enterobacteriaceae*
ICU: Intensive care unit
MIC: Minimal inhibitory concentration
